# PRDM16 functions as a suppressor of lung adenocarcinoma metastasis

**DOI:** 10.1186/s13046-019-1042-1

**Published:** 2019-01-25

**Authors:** Liang-Ru Fei, Wen-Jing Huang, Yuan Wang, Lei Lei, Zhi-Han Li, Yi-Wen Zheng, Zhao Wang, Mai-Qing Yang, Chen-Chen Liu, Hong-Tao Xu

**Affiliations:** Department of Pathology, College of Basic Medical Sciences and the First Affiliated Hospital, China Medical University, No.77 Puhe Road, Shenyang North New Area, Shenyang, Liaoning Province 110122 People’s Republic of China

**Keywords:** PRDM16, MUC4, EMT, Lung cancer

## Abstract

**Background:**

The transcription factor PR domain containing 16 (PRDM16) is known to play a significant role in the determination and function of brown and beige fat. However, the role of PRDM16 in tumor biology has not been well addressed. Here we investigated the impact of PRDM16 on tumor growth and metastasis in lung cancer.

**Methods:**

UALCAN database, immunoblotting and immunohistochemistry analysis were used to assess PRDM16 expression in lung cancer patients. Kaplan-Meier plotter database was used to analyze the overall survival of patients with lung cancer stratified by PRDM16 expression. PRDM16 overexpression and knockdown experiments were conducted to assess the effects of PRDM16 on growth and metastasis in vitro and in vivo, and its molecular mechanism was investigated in lung adenocarcinoma cells by chromatin immunoprecipitation-sequencing (ChIP-Seq), real time-quantitative PCR (RT-qPCR), luciferase assay, xenograft models and rescue experiments.

**Results:**

PRDM16 was downregulated in lung adenocarcinomas, and its expression level correlated with key pathological characteristics and prognoses of lung adenocarcinoma patients. Overexpressing PRDM16 inhibited the epithelial-to-mesenchymal transition (EMT) of cancer cells both in vivo and in vitro by repressing the transcription of Mucin-4 (MUC4), one of the regulators of EMT in lung adenocarcinomas. Furthermore, deleting the PR domain from PRDM16 increased the transcriptional repression of MUC4 by exhibiting significant differences in histone modifications on its promoter.

**Conclusions:**

Our findings demonstrate a critical interplay between transcriptional and epigenetic modifications during lung adenocarcinoma progression involving EMT of cancer cells and suggest that PRDM16 is a metastasis suppressor and potential therapeutic target for lung adenocarcinomas.

**Electronic supplementary material:**

The online version of this article (10.1186/s13046-019-1042-1) contains supplementary material, which is available to authorized users.

## Background

As a member of the PRDI-BF1 and RIZ domain-containing (PRDM) protein family, PRDM16 is structurally characterized by the combination of a conserved N-terminal PR domain and a variable number of zinc fingers [1–3]. The PR domain is similar to the suppressor of variegation 3–9, enhancer of zeste, and trithorax (SET) domain that defines a group of histone methyltransferases (HMTs) [1–3]. PRDM16 has been demonstrated to possess intrinsic histone methyltransferase activity and catalyze mono-methylation of histone 3 at lysine (H3K9me1) [4]. Therefore, PRDM16 may also act as a transcriptional regulator through its intrinsic chromatin-modifying activity or by complexing with histone-modifying enzymes [5]. PRDM16 regulates multiple biological processes including the determination and maintenance of brown adipocytes [6, 7], the switch between skeletal myoblasts and brown adipocytes [8–10], as well as the hematopoiesis and cardiac development [11, 12]. Human PRDM16 have four isoforms. Among them, two extensively studied isoforms are the full-length PRDM16 and the PR-lacking isoform generated by alternative promoter usage [13]. Notably, the PR-containing and PR-lacking isoforms of PRDM members sometimes exert opposing effects on tumor occurrence and development [2]. Compared with other PRDM members, the role of PRDM16 in cancer biology has been poorly studied and remains to be fully elucidated.

EMT is a fundamental biological process during normal organ development, tissue remodeling and wound healing [14]. In cancer biology, this complex phenotypic conversion determines the aggressiveness of cancer cells. During EMT process, cancer cells lose their cell polarity and adhesion, and gain the migratory and invasive capabilities of mesenchymal cells [14]. Therefore, EMT provides the basis and potential mechanisms for tumor progression and metastasis. Besides conventional epithelial and mesenchymal markers, several other important tumor antigens/mucins are also involved in EMT process. Among those, high molecular weight glycoprotein MUC4 plays a significant role in the cellular transformation and signaling alteration in the EMT process [15]. MUC4 belongs to the membrane-anchored class of mucins and has been reported to elevate in multiple epithelial malignancies [16, 17]. Recent studies have shown that MUC4 promotes cancer progression involving EMT in various human cancers, such as cervical cancer [18], ovarian cancer [19], pancreatic cancer [20, 21] and breast cancer [22]. Thus, MUC4 could be a potential therapeutic target for epithelial malignancies.

In this study, we showed that PRDM16 was downregulated in human lung adenocarcinomas, and its low expression predicted high lymph node status, TNM stage grade, and poor prognosis of lung adenocarcinoma patients. Overexpression of PRDM16 inhibited the cancer cell EMT process by suppressing the transcription of its target gene MUC4, which promoted EMT in lung adenocarcinomas. In addition, deleting the PR domain increased the transcriptional repression of MUC4 by reducing enrichment of H3K9me1 and acetylation of histone 3 at lysine (H3K9ac) on its promoter. Together, these results demonstrate that PRDM16 functions as a suppressor of tumor metastasis in human lung adenocarcinomas.

## Materials

### Cell culture, plasmid construction and transfection

All cell lines were obtained from the Shanghai Cell Bank (Shanghai, China), and cultured according to the instructions of the ATCC/CTCC. All cell lines were authenticated by short tandem repeat DNA profiling. G418 (#A1720, Sigma) was used to screen stably transfected cells. Lipofectamine 3000 (Invitrogen) transfection reagent was used for plasmid transfection. pCMV6-Myc-DDK (#PS100001) and pCMV6-Myc-DDK-PRDM16 (#RC214026) were from Origene (Rockville, MD, USA). PRDM16 deletion mutants were subcloned from pCMV6-Myc-DDK-PRDM16. The open reading frames of human MUC4 was cloned into the eukaryotic expression vector pCMV-C-HA (Beyotime, China). The shRNA plasmids were from from Genechem (Shanghai, China). The oligonucleotides were:

MUC4-shRNA-1#: 5’-TGTGAATTACTGCTACAAT-3’.

MUC4-shRNA-2#: 5’-CGCAAGCATCGGACTTCAC-3’.

MUC4-shRNA-3#: 5’-CAGCGACACTAGAGGGACA-3’.

PRDM16-shRNA-1#: 5’-CAATAGTGAGATGAACCAA-3’.

PRDM16-shRNA-2#: 5’-CCCACAACTTGCTGGTCAA-3’.

PRDM16-shRNA-3#: 5’-ACGGTGAAGCCTTTCATAT-3’.

MUC4/PRDM16-shRNA-Con: 5’-TTCTCCGAACGTGTCACGT-3’.

### Patients and specimens

All tumor specimens were obtained from surgical resection, and all patients were chemotherapy-naive and radiotherapy-naive prior to resection.

#### Primary tumor specimens for immunoblotting analysis

Primary lung cancer (37 cases lung adenocarcinoma, 23 cases lung squamous cell carcinoma) and matched normal lung tissue specimens (> 5 cm distal to the primary tumor’s edge) were obtained from 60 patients who underwent surgical resection at the First Affiliated Hospital of China Medical University. Samples were cut from the resected lungs immediately after removal, frozen in liquid nitrogen, and then stored at − 80 °C.

#### Paraffin sections

We collected 105 cases lung adenocarcinoma specimens from patients who underwent surgery at the First Affiliated Hospital of China Medical University from 2012 to 2014. All tissue specimens were fixed in neutral formaldehyde, embedded in paraffin, and sectioned to a thickness of 4 μm.

### Immunoblotting, immunofluorescence, and immunohistochemistry

Assays were performed as described previously [23]. For immunoblotting analysis, expression was quantified by the use of densitometry and ImageJ software. For immunofluorescence staining, cells were fixed, permeabilized and incubated with primary antibodies and fluorescein isothiocyanate-conjugated or tetramethylrhodamine isothiocyanate-conjugated secondary antibodies. The nuclei were counterstained with propidium iodide (PI, 50 μg/ml, Sigma), and cells were observed under a confocal microscope. For immunohistochemistry staining, the intensity of staining was scored as follows: 0 (no staining), 1 (weak), 2 (moderate), or 3 (high). Percentage scores were assigned as follows: 1 (1–25%), 2 (26–50%), 3 (51–75%), and 4 (76–100%). The scores of each tumor sample were multiplied to give a final score of 0–12, and tumor samples with scores of ≧6 were considered to have positive expression, those with scores ≧ 0 and < 6 were considered to have negative expression. Information on primary antibodies is provided in Additional file 1: Supplementary Materials and Methods.

### MTT and colony formation assays

Assays were performed as described previously [23]. For colony formation assay, the number of colonies with more than 50 cells was counted. For MTT assay, the measurement process was performed every 24 h for 5 days to generate a cell growth curve. All experiments were performed in triplicate.

### Matrigel invasion and wound healing assays

Cell invasion assay was performed using a 24-well Transwell chamber with a pore size of 8 μm (Costar), and the inserts were coated with 20 μl Matrigel (1:3 dilution, BD Bioscience). Forty eight hours after transfection, we added 6 × 105 cells in 100 μl medium supplemented with 2% FBS to the upper Matrigel chamber and incubated for 20 h. Medium supplemented with 20% FBS was added to the lower chamber as the chemoattractant. The numbers of invaded cells were counted in 10 randomly selected high-power fields under a microscope. For the wound healing assay, cells were plated to confluence in a 6-well plate, and the cell monolayer was scratched using a pipette tip. Wound healing within the scrape line was observed at indicated time points, and representative scrape lines for each cell line were photographed. Duplicate wells for each condition were examined for each experiment. The distance of the wound was optically measured using Image J software. All experiments were performed in triplicate.

### Dual-luciferase assay

Luciferase reporter construct of MUC4 promoter (2.5 kb upstream of transcription start site (TSS)) was co-transfected with indicated plasmids, together with Renilla luciferase as a control for signal normalization. Dual luciferase assays were performed according to the manufacturers protocol (Promega). Three independent transfections were carried out for each experiment. Data were normalized to the empty vector control and presented as average ± SD.

### RNA extraction and real time-quantitative PCR (RT-qPCR)

Assays were performed as described previously [24]. The relative transcript levels of genes were normalized to GAPDH mRNA levels, and the primer sequences are shown in Additional file 1: Supplementary Materials and Methods.

### Tumor xenograft mouse models

The animal study was approved by the Institutional Animal Research Committee of China Medical University. All procedures were in agreement with experimental animal ethics guidelines issued by China Medical University. Four-week-old female BALB/c nude mice were purchased from Charles River (Beijing, China), and the axilla or tail vein of each mouse was subcutaneously or intravenously inoculated with 5 × 106 or 1 × 106 tumor cells, respectively, in 0.2 ml of sterile PBS. Six weeks after inoculation, the mice were sacrificed and autopsied to examine tumor growth and dissemination. A portion of tissue from the tumor and lung was fixed in 4% formaldehyde (Sigma) and embedded in paraffin. Serial 4-μm-thick sections were cut and conducted with haematoxylin and eosin (HE) or immunohistochemistry staining analysis .

### Chromatin immunoprecipitation (ChIP)

Cells were fixed in 1% formaldehyde for cross-linking of chromatin. A standard ChIP assay was performed according to the ChIP assay kit protocol (Millipore). Normal rabbit IgG served as the control. Immunoprecipitated DNA enrichment was normalized to its Input. Antibodies used were: Myc-tag (#2276, Cell Signaling Technology), PRDM16 (#ABE543, Millipore), H3K9me1 (#14186, Cell Signaling Technology), H3K9me2 (#4658, Cell Signaling Technology), H3K9me3 (#13969, Cell Signaling Technology), H3K9ac (#9649, Cell Signaling Technology). For ChIP-sequencing (ChIP-Seq), libraries for sequencing were obtained using the ChIP-seq DNA sample prep kit (Illumina) according to manufacturer recommendations and samples were sequenced on a Genome Analyzer II sequencer (Illumina). For ChIP-qPCR, primers used are provided in Additional file 1: Supplementary Materials and Methods.

### Statistics

The immunohistochemistry results were analyzed using the chi-square test and Spearman rank correlation. Differences between groups were compared using a two-tailed Student’s t test and considered significant when *p* values were < 0.05(*) or < 0.01(**).

## Results

### Low PRDM16 expression is associated with poor prognosis of lung adenocarcinoma patients

**Fig. 1:**
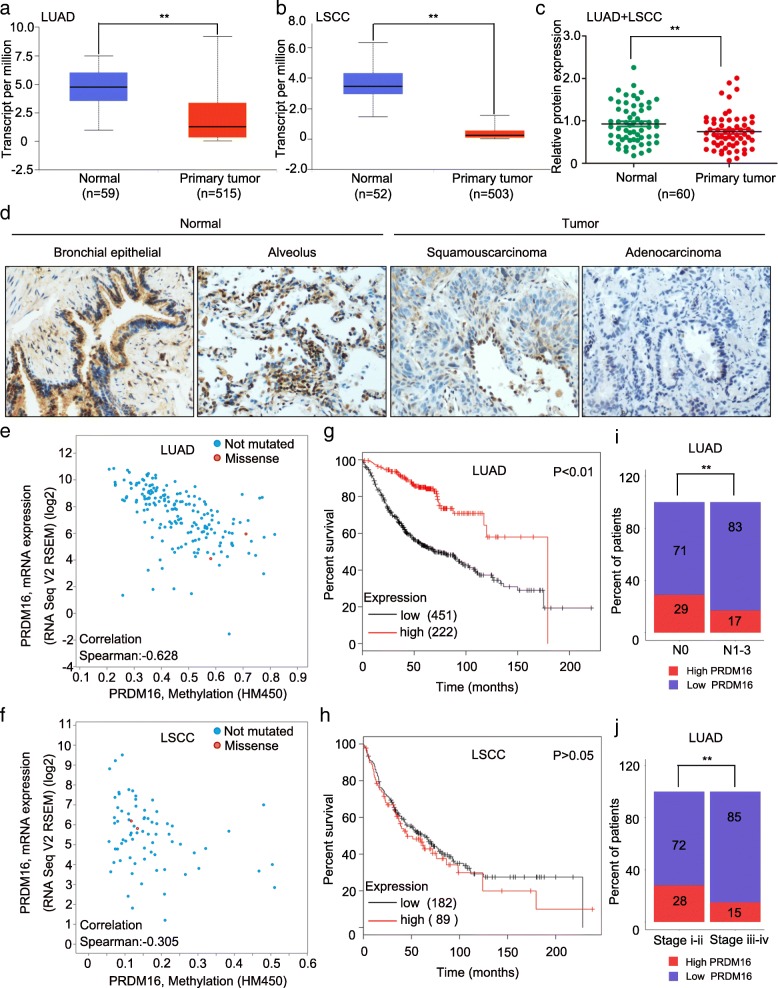
Downregulation of PRDM16 correlates with poor prognosis of lung adenocarcinoma patients. **a** and **b**, Transcriptional level of PRDM16 in lung adenocarcinoma (LUAD) and lung squamous cell carcinoma (LSCC) compared to that in normal lung tissues. Data were obtained from the UALCAN database [35]. **c**, Immunoblotting analysis of PRDM16 expression in lung cancer and matched normal lung tissues (magnification: × 200). β-actin was quantified as an internal control. **d**, Immunohistochemistry staining of PRDM16 in lung cancer and normal lung tissues. **e** and **f**, Correlation scatter plot of the PRDM16 mRNA expression and methylation levels in lung adenocarcinoma and lung squamous cell carcinoma. Data were obtained from the cBioPortal database [36, 37]. **g** and **h**, Kaplan-Meier plots of overall survival of patients with lung adenocarcinoma and lung squamous cell carcinoma stratified by PRDM16 expression. Data were obtained from the Kaplan-Meier plotter database [38]. **i** and **j**, Percentage of lung adenocarcinoma patients with high expression and low expression of PRDM16 stratified according to lymph node status or TNM stage (*n* = 497)

To assess PRDM16 expression in lung cancer patients, we first analyzed the gene expression datasets of human lung adenocarcinomas and lung squamous cell carcinomas. The results showed that PRDM16 mRNA level in lung adenocarcinoma and lung squamous cell carcinoma tissues were significantly lower than that in normal lung tissues (Fig. 1a and b). Consistent with the mRNA level, PRDM16 protein expression was also significantly downregulated in both lung adenocarcinomas and lung squamous cell carcinomas compared with normal lung tissues (Fig. 1c and d). Furthermore, downregulated PRDM16 expression may result from the high methylation levels of its DNA in lung adenocarcinomas but not in lung squamous cell carcinomas (Fig. 1e and f). We performed Kaplan-Meier survival analysis to determine whether PRDM16 expression predicts patient outcome. Interestingly, we found only lung adenocarcinoma patients with low PRDM16 expression had shorter overall survival (Fig. 1g and h). Then we used The Cancer Genome Atlas (TCGA) gene expression and clinical data to further determine whether PRDM16 is associated with other clinical and pathological characteristics of lung adenocarcinoma patients. Notably, the analysis indicated that low PRDM16 expression was correlated with higher lymph node status and TNM stage grade in lung adenocarcinoma patients (Fig. 1i and j). Taken together, these results indicated that PRDM16 expression level is downregulated in human lung adenocarcinoma tissues and correlated with poor prognosis of lung adenocarcinomas.

### PRDM16 inhibits lung adenocarcinoma cell migration and invasion in vitro

**Fig. 2:**
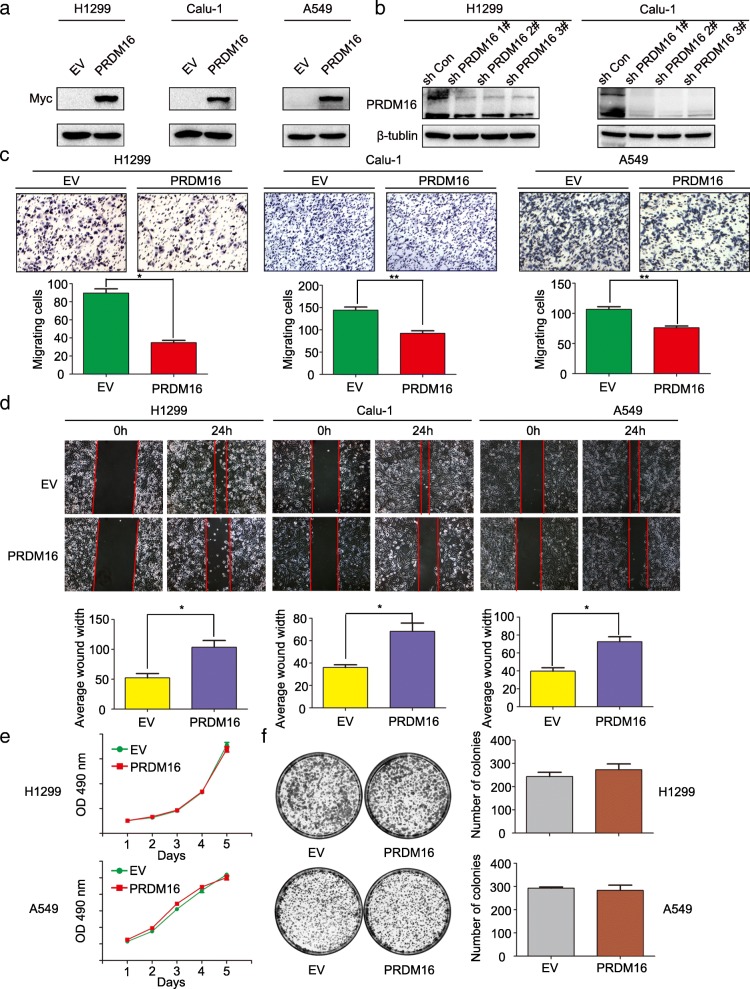
Effect of PRDM16 expression on the invasiveness and proliferation of lung adenocarcinoma cells in vitro. **a**, Immunoblotting analysis of PRDM16 expression in H1299, Calu-1 and A549 cells treated with Myc-DDK-PRDM16 (PRDM16) or empty vector control (EV). **b**, Immunoblotting analysis of PRDM16 expression in H1299 and Calu-1 cells treated with shRNAs specific for PRDM16 (sh PRDM16 1#, sh PRDM16 2# and sh PRDM16 3#) or scramble control (sh Con). **c** and **d**, Images and quantitation of matrigel invasion assays or wound healing assays of H1299, Calu-1 and A549 cells treated with EV and PRDM16. **e** and **f**, MTT assays or colony formation assays for H1299 and A549 cells treated with EV and PRDM16

**Fig. 3:**
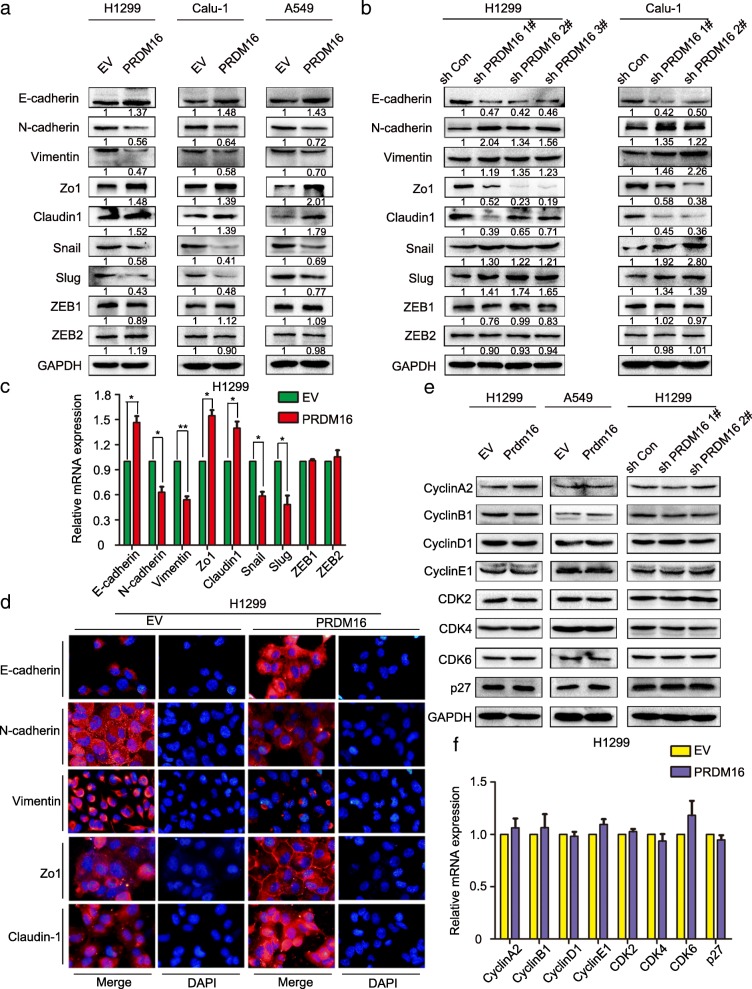
Effect of PRDM16 expression on key EMT markers and cell cycle regulators of lung adenocarcinoma cells in vitro. **a** and **b**, Immunoblotting analysis of expression of key EMT-related markers in H1299, Calu-1 and A549 cells treated with indicated plasmids. GAPDH was used as an internal control. The relative protein level of control group was used to normalize protein levels in other groups. **c**, RT-qPCR analysis of mRNA levels of indicated key EMT-related genes in EV and PRDM16 cells normalized to GAPDH. **d**, Immunofluorescence staining of indicated key EMT-related markers in EV and PRDM16 cells. **e**, Immunoblotting analysis of expression of indicated key cell cycle regulators in H1299 and A549 cells treated with indicated plasmids. **f**, RT-qPCR analysis of mRNA levels of indicated key cell cycle regulators in EV and PRDM16 cells normalized to GAPDH

To investigate the effect of PRDM16 on cell growth and mobility in lung adenocarcinoma, we overexpressed PRDM16 in H1299, Calu-1 and A549 cells (Fig. 2a) or knocked down its expression in H1299 and Calu-1 cells (Fig. 2b). As compared with the control cells, PRDM16 overexpression inhibited and PRDM16 knockdown promoted cell migration and invasion (Fig. 2c and d; Additional file 2: Figure S1a and b), but hardly affected cell proliferation (Fig. 2e and f). Then, how does PRDM16 regulate the migratory and invasive phenotypes of cancer cells? We found that PRDM16 overexpression inhibited and PRDM16 knockdown promoted the EMT progression of lung adenocarcinoma cells by regulating the expression of epithelial and mesenchymal markers (Fig. 3a and b). Interestingly, in terms of transcription factors, the expression of Snail and Slug was significantly regulated by PRDM16, whereas the expression of ZEB1, or ZEB2 was not affected (Fig. 3a and b). Moreover, real time-quantitative PCR (RT-qPCR) and immunofluorescence staining experiments also confirmed that epithelial markers were significantly increased, whereas mesenchymal markers decreased in PRDM16 overexpressed cells compared to that in the control cells (Fig. 3c and d). In contrast, the epithelial markers were decreased, whereas mesenchymal markers increased in PRDM16 knockdown cells (Additional file 2: Figure S1c). To further verify whether PRDM16 affects cell growth and proliferation, we examined some key regulators during cell cycle progression both in protein and mRNA levels. In accordance with the results from functional analysis, PRDM16 hardly affected cell growth (Fig. 3e and f). Thus, these results suggest that PRDM16 is a crucial regulator for lung cancer cell migration and invasion in vitro.

### PRDM16 inhibits lung adenocarcinoma cell metastasis in vivo

**Fig. 4:**
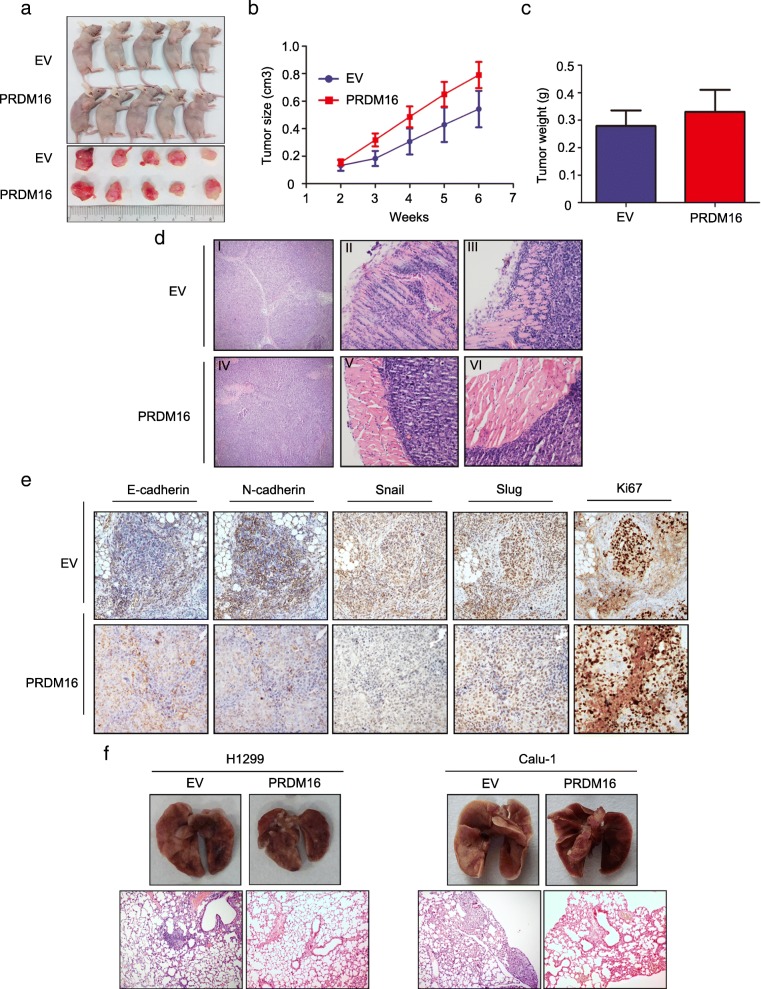
Effect of PRDM16 expression on the proliferation and invasiveness in xenograft mouse models. **a**, **b** and **c**, Subcutaneous injection of H1299 cells stably expressing EV or PRDM16 (screened by G418) into nude mice. The average tumor volume (*p* = 0.27) and weight (*p* = 0.19) were quantified. **d**, HE staining of tumor tissue of mice subcutaneously injected with H1299-EV and H1299-PRDM16 cells. **e**, Immunohistochemistry analysis of indicated key EMT-related markers and Ki67 in tumor tissue of mice subcutaneously injected with H1299-EV or H1299-PRDM16 (magnification: × 200). **f**, Pulmonary metastases of mice intravenously injected with H1299-EV/PRDM16 and Calu1-EV/PRDM16 cells

To investigate the biological function of PRDM16 in vivo, a H1299 cell line stably expressing PRDM16 was subcutaneously injected into nude mice. Although the tumor formation rate (5/5, 100%), volume and weight of subcutaneously injected tumors were similar (Fig. 4a, b and c), the invasive ability of cancer cells was decreased in the PRDM16 overexpression group (Fig. 4d). The cancer cells showed multinodular growth (Fig. 4d-I) and sharply infiltrated into the normal tissues (Fig. 4d-II and III) in the EV group. But in the PRDM16 overexpression group, the cancer cells showed mononodular growth (Fig. 4d-IV), and the edge of the cancer cell nest was blunt and well demarcated (Fig. 4d-V and VI). Additionally, immunohistochemistry staining of E-cadherin, N-cadherin, Snail and Slug indicated a lower EMT status in cancer cells overexpressing PRDM16 compared to that in the control group, whereas the Ki67 staining was similar between the two groups (Fig. 4e). Then, we injected H1299 and Calu-1 cells stably expressing PRDM16 intravenously into nude mice via the tail vein. Compared with the intrapulmonary metastasis formation rate of control groups (H1299 cell line: 3/5; Calu-1 cell line: 4/5), there was no intrapulmonary metastasis in both two PRDM16 overexpressing groups (Fig. 4f). Collectively, these data indicate that PRDM16 functions as a key regulator in inhibiting the metastasis of lung adenocarcinoma cells in vivo.

### Overexpressing PRDM16 suppresses cancer cell EMT by downregulating MUC4

**Fig. 5:**
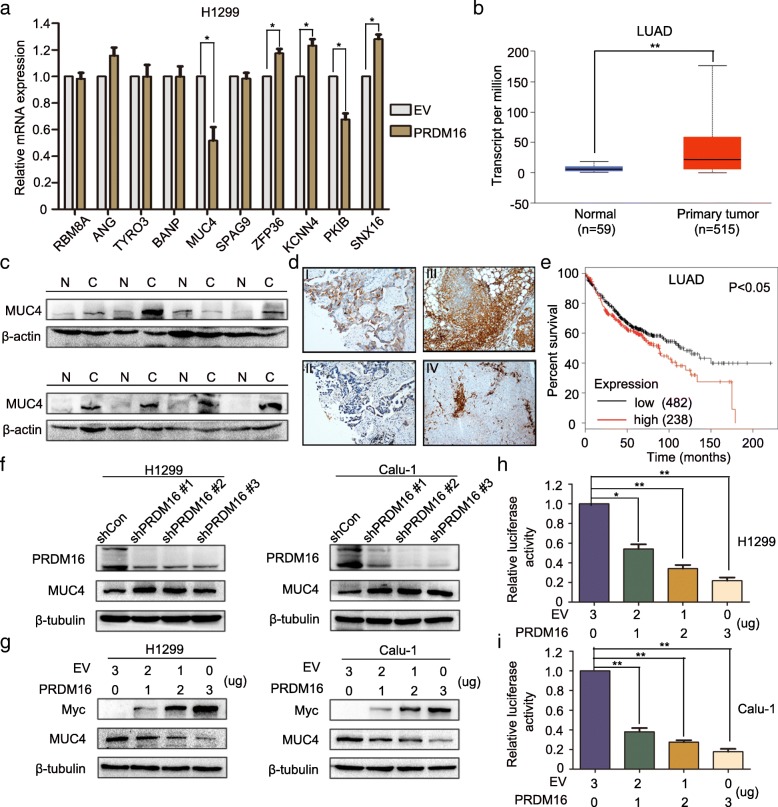
PRDM16 functions as a MUC4 transcriptional repressor. **a**, qPCR analysis of validation PRDM16 target genes from the ChIP-Seq experiment in H1299 cells transfected with EV or PRDM16. **b**, Transcription level of MUC4 in lung adenocarcinoma compared with normal lung tissues. Data were obtained from the UALCAN database [37]. **c**, Immunoblotting analysis of MUC4 expression in eight randomly chosen lung adenocarcinoma tissues (C) and matched normal lung tissues (N). **d**, Immunohistochemistry staining of MUC4 (I) and PRDM16 (II) in the same human lung adenocarcinoma tissue, and MUC4 staining in tumor tissues of mice subcutaneously injected with H1299-EV (III) and H1299-PRDM16 (IV) (magnification: × 200). **e**, Kaplan-Meier plots of overall survival of patients with lung adenocarcinoma stratified by MUC4 expression. Data were obtained from the Kaplan-Meier plotter database [36]. **f** and **g**, Immunoblotting analysis of MUC4 in H1299 and Calu-1 cells treated with indicated plasmids. **h** and **i**, Dual-luciferase assays for the effect of PRDM16 on MUC4 transcriptional repression in H1299 and Calu-1 cells

**Fig. 6:**
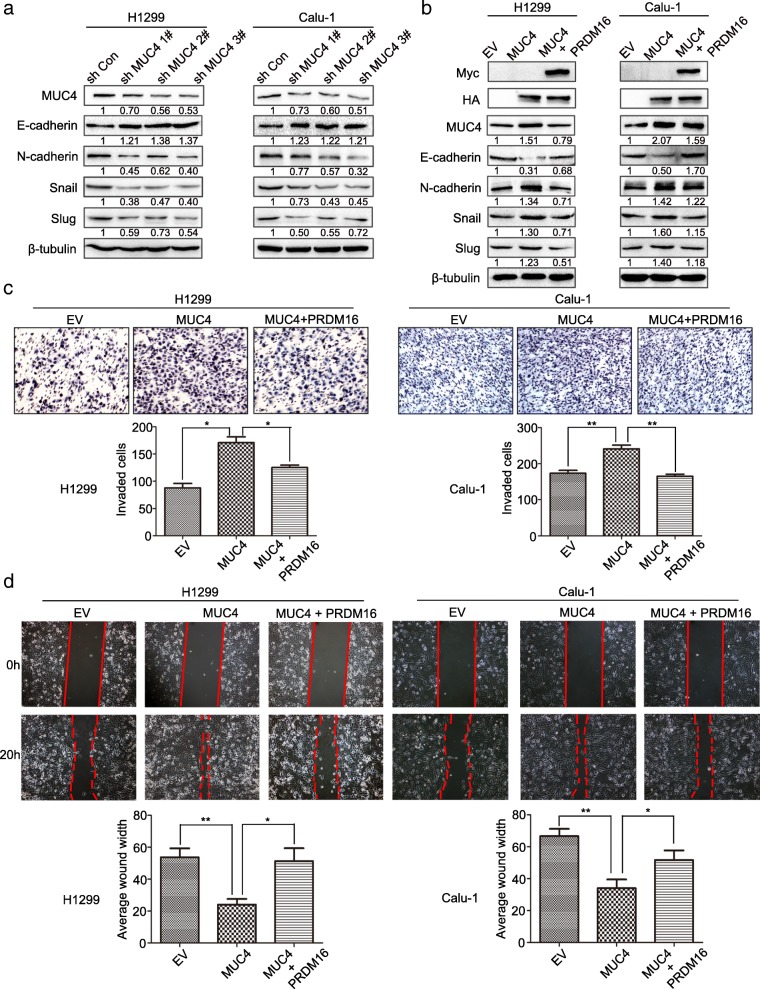
PRDM16 suppresses cancer cell EMT by downregulating MUC4 expression in lung adenocarcinomas. **a** and **b**, Immunoblotting analysis of expression of key EMT-related markers in H1299 and Calu-1 cells treated with indicated plasmids. **c** and **d**, Images and quantitation of matrigel invasion and wound healing assays of EV cells, MUC4 cells, and MUC4 cells with enforced PRDM16 expression (MUC4 + PRDM16)

Given that PRDM16 has been reported to function mainly as a transcriptional regulator [5], we next performed ChIP-sequencing (ChIP-Seq) to identify direct PRDM16 target genes and further investigate the mechanism through which it inhibits cancer cell EMT. In order to more efficiently identify PRDM16 target genes, H1299 cells stably expressing PRDM16 (Myc-DDK-PRDM16) were subjected to ChIP assays with two antibodies, Myc-tag and PRDM16, for analysis (Additional file 3: Table S1). Based on the mutual genes between the two groups, we screened the ones closely associated with EMT, and performed qPCR to further confirm the significant target genes of PRDM16 (Fig. 5a). Among the candidate target genes, MUC4 was elevated in lung adenocarcinoma tissues (Fig. 5b and c), and its expression negatively correlated with PRDM16 (Fig. 5d and Table 1). In addition, high MUC4 expression was associated with poor prognosis of lung adenocarcinoma patients (Fig. 5e). These data suggest that PRDM16 may function as a MUC4 transcriptional repressor. To validate this, we knocked down PRDM16 or overexpressed PRDM16 in a dose-dependent manner in H1299 and Calu-1 cells. As compared with the control group, MUC4 expression was upregulated by PRDM16 knockdown, and downregulated by PRDM16 overexpression in a dose-dependent manner (Fig. 5f and g). Luciferase reporter assay also demonstrated that PRDM16 inhibited the transcription of MUC4 (Fig. 5h and i). Then, we knocked down or overexpressed MUC4 in H1299 and Calu-1. Compared with the control group, low MUC4 expression suppressed, while high MUC4 expression promoted, the EMT process (Fig. 6a and b). Then, we tested the possibility that enforced expression of PRDM16 would compensate for MUC4 overexpression. As expected, the suppression of E-cadherin and increased expression of N-cadherin, Snail and Slug induced by elevated MUC4 expression were restored by ectopic expression of PRDM16 (Fig. 6b), which was accompanied by a similar effect on migratory and invasive capabilities (Fig. 6c and d). Taken together, PRDM16 suppresses cancer cell EMT by downregulating MUC4 expression in lung adenocarcinoma cells.

**Table 1:** Correlation between PRDM16 and MUC4 expression in lung adenocarcinomas

MUC4 expression					
		Positive	Negative		
		*N*		R	*P* value
PRDM16 expression	Positive	12	21	−0.506	< 0.001
	Negative	62	10

### PRDM16-mediated transcriptional repression of MUC4 primarily depends on DNA-binding domain 2

**Fig. 7:**
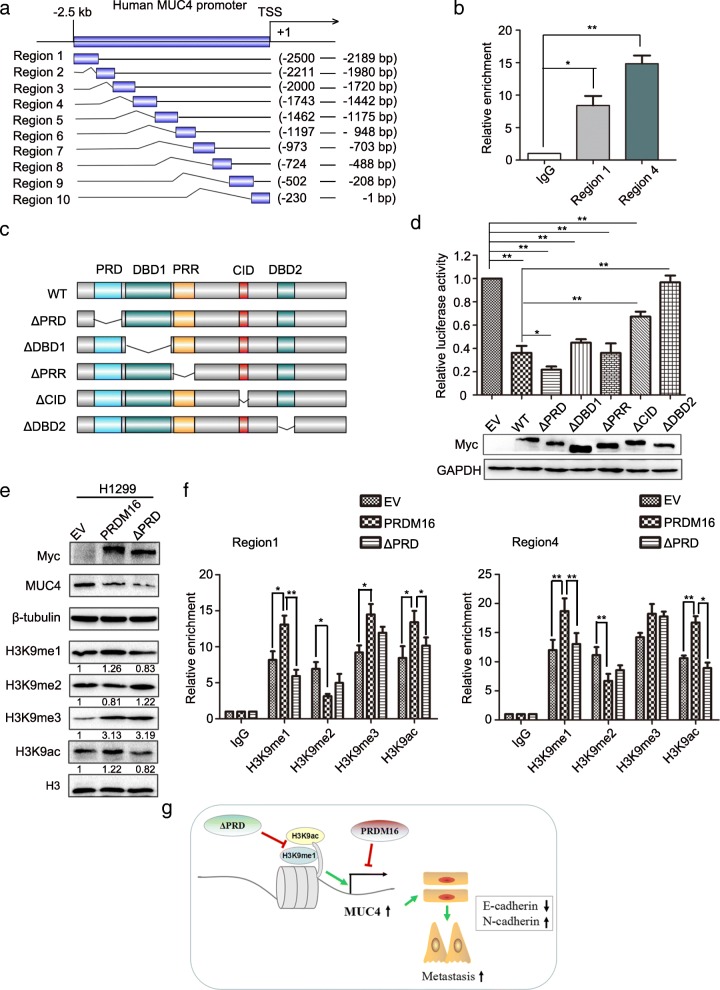
PRDM16-mediated transcriptional repression of MUC4. Deleting the PR domain of PRDM16 increases transcriptional repression of MUC4 by regulating histone modifications on the MUC4 promoter. **a**, Schematic illustration of ten regions in the MUC4 promoter. **b**, ChIP-qPCR assays for binding regions of PRDM16 on MUC4 promoter. **c**, Domain locations within the structure of PRDM16. **d**, Dual-luciferase assays for H1299 cells transfected with indicated plasmids. **e**, Immunoblotting analysis of indicated protein levels H1299 cells transfected with EV, PRDM16, PRDM16-ΔPRD. **f**, ChIP-qPCR analysis of the enrichment of indicated H3 modifications on Region 1 (left) and Region 4 (right) of the MUC4 promoter in H1299 cells transfected indicated plasmids. **g**, A proposed model to illustrate the role of PRDM16 in EMT. Overexpression of PRDM16 inhibits the EMT process by suppressing the transcription of its target gene MUC4. PRDM16-ΔPRD increases the transcriptional repression of MUC4 by reducing enrichment of H3K9me1 and H3k9ac on its promoter

To investigate the domains of MUC4 promoter binding with PRDM16, we divided 2.5 kb upstream of TSS MUC4 into ten parts (Fig. 7a) and further performed ChIP assay. The results indicated that PRDM16 bound to two regions of MUC4 promoter, Region 1 (− 2500 bp – -2189 bp) and Region 4 (− 1743 bp – -1442 bp) (Fig. 7b). To determine the domain(s) of PRDM16 that are responsible for transcriptional repression, we constructed a series of deletion mutants of PRDM16 and transfected full-length (wild-type, WT) and mutant PRDM16 plasmids into H1299 cells, and conducted luciferase reporter assays. These mutants represent deletions of PR domain (ΔPRD), DNA-binding domain 1 (ΔDBD1), the proline-rich region (ΔPRR), the C-terminal-binding protein (CtBP)-interacting domain (ΔCID) and DNA-binding domain 2 (ΔDBD2) (Fig. 7c). Transcriptional repression was clearly reduced with PRDM16-ΔCID and PRDM16-ΔDBD2. PRDM16-ΔDBD2 showed almost no repressor activity compared with PRDM16-WT. Therefore, the DBD2 may be the most important domain for the repressor activity of PRDM16 (Fig. 7d). Furthermore, an interesting finding was that PRDM16-ΔPRD increased transcriptional repression compared with PRDM16-WT (Fig. 7d).

### PRDM16-ΔPRD increases transcriptional repression of MUC4 by regulating histone modifications on the MUC4 promoter

The PR domain is usually closely related to the histone methyltransferase activity of PRDM members [1–3]. In addition, PRDM16 has been reported to catalyze H3K9me1 in immortalized mouse embryonic fibroblasts [4]. To determine whether the increased transcriptional repression of MUC4 by PRDM16-ΔPRD is associated with histone modifications, we overexpressed PRDM16-WT and PRDM16-ΔPRD in H1299 cells. Compared with PRDM16-WT overexpressing cells, the expression of H3K9me1 and H3K9ac was reduced and di-methylation of histone 3 at lysine (H3K9me2) expression was increased in PRDM16-ΔPRD overexpressing cells, whereas tri-methylation of histone 3 at lysine (H3K9me3) expression did not changed in this context (Fig. 7e). As histone modifications are demonstrated to play an important role in transcriptional regulation [25, 26], we further examined the enrichment of H3K9me1, H3K9me2, H3K9me3, and H3K9ac on the MUC4 promoter. Consistent with the pan-cellular western blot analysis, high PRDM16-ΔPRD overexpression significantly reduced H3K9me1 and H3K9ac enrichment, and had little effect on H3K9me3 enrichment on the MUC4 promoter compared to that in PRDM16-WT overexpressing cells. However, we did not detect a significant difference in H3K9me2 enrichment on the MUC4 promoter between PRDM16-ΔPRD and PRDM16-WT overexpressing cells (Fig. 7f). As both H3K9me1 and H3K9ac tend to correlate with active promoters [25, 26], these data may indicate that PRDM16-ΔPRD increases transcriptional repression by reducing H3K9me1 and H3K9ac enrichment on MUC4 promoter.

## Discussion

It has been reported that the short variant of PRDM16, PR-lacking PRDM16 was involved in several hematological malignancies through blocking differentiation of progenitor cells and promoting growth of leukemia cells. [13, 27, 28]. Yoshida et al. found that PR-lacking PRDM16, but not full-length PRDM16, repressed transforming growth factor β (TGF β)-mediated growth inhibition in adult T cell leukemia cells [27]. In contrast, Mami et al. reported that full-length PRDM16 interacted with SKI, a known repressor of TGF β signaling, to prevent TGF β-induced growth inhibition by stabilizing the inactive Smad3-SKI complex on the promoter of TGF β target genes in human gastric cancer cells [29]. Our study discovered that PRDM16 expression was downregulated in lung adenocarcinomas, and its low expression predicted poor prognosis of lung adenocarcinoma patients. Overexpression of full-length PRDM16 or PRDM16-ΔPRD can suppress the transcription of MUC4 which is demonstrated to promote EMT in lung adenocarcinomas, suggesting full-length and PR-lacking PRDM16 exert similar effects on the metastatic ability of cancer cell in lung adenocarcinomas. Besides, we found elevated PRDM16 expression inhibits cancer cell invasion and migration but hardly affects cell proliferation in lung adenocarcinomas. It is possible that PRDM16 plays different roles in different malignancies. Our findings implicate PRDM16 as an important negative regulator of metastasis in lung adenocarcinomas.

MUC4 has been reported to promote cancer progression involving EMT in a variety of epithelial carcinomas [18–22]. However, the role of MUC4 in the occurrence and development of lung cancer seems controversial. Prabin et al. [30] has reported that MUC4 plays a tumor-suppressor role in lung cancers, and high MUC4 expression was associated with a better overall survival in analysis of 29 patients with survival data. Gao et al. [31] reported that MUC4 suppresses EMT in lung adenocarcinomas by modulating β-catenin, and that MUC4 expression correlates with a risk of lymph node metastasis in a cohort of 20 lung adenocarcinoma patients. However, the studies with large size samples obtained different results. Tsutsumida et al. reported that high MUC4 expression correlates with a poor survival rate in a cohort of 185 lung adenocarcinomas patients [32]. Recently, Mariyo et al. found that high MUC4 expression is significantly associated with vascular invasion in 338 lung adenocarcinomas, and patients with MUC4-positive tumors had worse prognoses [33]. Here, we analyzed the gene expression datasets of human lung cancer and showed that MUC4 mRNA level in lung adenocarcinoma and lung squamous cell carcinoma tissues were significantly higher than that in normal lung tissues (Fig. 5b and Additional file 2: Figure S1d). But only lung adenocarcinoma patients with high MUC4 expression had shorter overall survival (Fig. 5e and Additional file 2: Figure S1e). However, the analysis results from TCGA datasets indicated that lung adenocarcinoma patients with high MUC4 expression did not significantly correlate with higher lymph node status and TNM stage grade in statistics (Additional file 2: Figure S1f and g). But our results in vitro showed that MUC4 promotes EMT in lung adenocarcinoma. Differences in cell lines, number of cases or patients may contribute to the inconsistency of results. More abundant data may provide more reliable results in future.

In this study, we showed that PRDM16 functions as a transcriptional repressor of MUC4 in lung adenocarcinomas. The transcriptional repression of MUC4 mediated by PRDM16 is closely associated with CID and DBD2 of PRDM16. Nishikata et al. reported that the transcriptional repressor activity of PR-lacking PRDM16 is mainly mediated by sumoylation and its interaction with CtBP through the CID, which contributes to the development of leukemia [34]. Here, PRDM16-ΔCID still repressed the transcription of MUC4, suggesting other domains were involved. Compared with PRDM16-ΔCID, PRDM16-ΔDBD2 showed completely abrogated repressor activity of PRDM16, indicating that DBD2 was the most important domain for the repression of MUC4. Since DBD2 is a series of zinc-finger repeats which is used to bind with DNA, PRDM16 may bind to the MUC4 promoter by DBD2, thus hindering the loading of other transcriptional activators on the MUC4 promoter.

In addition, we found that PRDM16-ΔPRD increased the repression of MUC4 compared with full-length PRDM16 by reducing H3K9me1 and H3K9ac enrichment on the MUC4 promoter. It is well known that aberrant epigenetic signatures are associated with abnormal developmental processes and diseases such as cancer [26]. Histone modification is the one of the most important mechanisms for epigenetic regulation. Among those, acetylation of histones is thought to relax condensed heterochromatin as the negative charge of acetyl groups can antagonize the DNA phosphate backbone charges, thus reducing the histone binding affinity for DNA and promoting gene activation [26]. Histone methylation, especially on the lysine residues in the N-terminal of histone 3, can either activate or repress gene transcription, depending on the site of lysine and the number of methyl groups [25, 26]. For example, H3K9me1 is generally considered as a gene expression activation mark, whereas H3K9me2 and H3K9me3 are both found more often enriched at silenced genes [25, 26]. Here, we found that the levels of H3K9me1/2/3 and H3K9ac in total cellular lysates and on MUC4 promoter were changed significantly in PRDM16-WT overexpressing cells compared with EV cells (Fig. 7e and f). Alterations in these histone modification markers between the two groups may be correlated with the intrinsic histone methyltransferase activity of PRDM16, and/or the interaction between PRDM16 and other histone-modifying enzymes. However, we could not find out the specific effect of these histone modifications on the repression of MUC4 mediated by PRDM16. What we can make sure is that MUC4 transcription was significantly repressed in PRDM16 overexpressing cells compared with EV cells. Nonetheless, the role of histone modification in gene transcription depends on numerous factors which might form a complex network and still needs further study.

## Conclusions

Taken together, our study elucidates a novel mechanism of PRDM16 inhibiting the EMT and metastasis of lung adenocarcinoma cells (Fig. 7g). As well as highlighting PRDM16 as a potential biomarker and drug target in lung adenocarcinomas, these data also demonstrate a critical interplay between transcriptional and epigenetic modifications during lung adenocarcinoma progression.

## Additional files

Additional file 1: Supplementary Materials and Methods. (DOC 54 kb)

Additional file 2: **Figure S1.** (ZIP 4948 kb)

Additional file 3: **Table S1.** ChIP data. (XLS 2153 kb)

## Abbreviations

ChIP-Seq: Chromatin immunoprecipitation-sequencing
EMT: Epithelial-to-mesenchymal transition
H3K9ac: Acetylation of histone 3 at lysine
H3K9me1/2/3: Mono/di/tri-methylation of histone 3 at lysine
HMTs: Histone methyltransferases
LSCC: Lung squamous cell carcinoma
LUAD: Lung adenocarcinoma
MUC4: Mucin-4
PRDM: PRDI-BF1 and RIZ domain-containing protein
PRDM16: Transcription factor PR domain containing 16
RT-qPCR: Real time-quantitative PCR
SET: Suppressor of variegation 3–9, enhancer of zeste, and trithorax
TCGA: The Cancer Genome Atlas
TGF β: Transforming growth factor β
TSS: Transcription start site
