# Supplementary material for: PRDM16 functions as a suppressor of lung adenocarcinoma metastasis
Source: J Exp Clin Cancer Res. 2019 Jan 25;38:35. doi: 10.1186/s13046-019-1042-1 (PMC6347838; doi:10.1186/s13046-019-1042-1)
Supplement: Supplementary file 1 — Supplementary Materials and Methods. (DOC 54 kb) [file 13046_2019_1042_MOESM1_ESM.doc]

**Additional file 1:** Supplementary Materials and methods

**Immunoblotting, immunofluorescence, and immunohistochemistry**

Immunoblotting (IB), immunofluorescence (IF), and immunohistochemistry (IHC) were carried out with the following primary antibodies: Myc-tag (#2276, Cell Signaling Technology, IB/1:1000), PRDM16 (#ABE543, Millipore, IB/1:1000, IHC/1:100), GAPDH (#60004-1-Ig, Proteintech, IB/1:1000 ), β-tubulin (#10094-1-AP, Proteintech, IB/1:1000 ), E-cadherin (#14472, Cell Signaling Technology, IB/1:750, IHC/1:50, IF/1:50), N-cadherin (#13116, Cell Signaling Technology, IB/1:1000, IHC/1:50, IF/1:50), Vimentin (#5741, Cell Signaling Technology, IB/1:1000, IF/1:50), Zo1 (#13663, Cell Signaling Technology, IB/1:1000, IF/1:50), Claudin1 (#13995, Cell Signaling Technology, IB/1:1000, IF/1:50), Snail (#13099-1-AP, Proteintech, IB/1:1000, IHC/1:100), Slug (#12129-1-AP, Proteintech, IB/1:750, IHC/1:100), ZEB1 (#3396, Cell Signaling Technology, IB/1:1000), ZEB2 (#14026-1-AP, Proteintech, IB/1:1000), CyclinA2 (#4656, Cell Signaling Technology, IB/1:750), CyclinB1 (#4138, Cell Signaling Technology, IB/1:1000), CyclinD1 (#2978, Cell Signaling Technology, IB/1:500), CyclinE1 (#4129, Cell Signaling Technology, IB/1:750), CDK2 (#2546, Cell Signaling Technology, IB/1:1000), CDK4 (#12790, Cell Signaling Technology, IB/1:1000), CDK6 (#13331, Cell Signaling Technology, IB/1:1000), p27 (#3686, Cell Signaling Technology, IB/1:500), MUC4 (#55343-1-AP, Proteintech, IB/1:1000), MUC4 (#ab150381, Abcam, IHC/1:50), β-actin (#60008-1-Ig, Proteintech, IB/1;1000), H3K9me1 (#14186, Cell Signaling Technology, IB/1:1000), H3K9me2 (#4658, Cell Signaling Technology, IB/1:1000), H3K9me3 (#13969, Cell Signaling Technology, IB/1:1000), H3K9ac (#9649, Cell Signaling Technology, IB/1:750), H3 (#14269, Cell Signaling Technology, IB/1:1000).

**RNA extraction and RT-qPCR**

Primers used were:

| E-cadherin: | 5’-ATTTTTCCCTCGACACCCGAT-3’ (forward)  5’-TCCCAGGCGTAGACCAAGA-3’ (reverse) |
| --- | --- |
| N-cadherin: | 5’-TCAGGCGTCTGTAGAGGCTT-3’ (forward)  5’-ATGCACATCCTTCGATAAGACTG-3’ (reverse) |
| Vimentin: | 5’-TGCCGTTGAAGCTGCTAACTA-3’ (forward)  5’-CCAGAGGGAGTGAATCCAGATTA-3’ (reverse) |
| Zo1: | 5’-CAACATACAGTGACGCTTCACA-3’ (forward)  5’-CACTATTGACGTTTCCCCACTC-3’ (reverse) |
| Claudin1: | 5’-AGCTGCAAAATGTACGACTCG-3’ (forward)  5’-GGAGACCACCATTAGGGCTC-3’ (reverse) |
| Snail: | 5’-ACTGCAACAAGGAATACCTCAG-3’ (forward)  5’-GCACTGGTACTTCTTGACATCTG-3’ (reverse) |
| Slug: | 5’-CGAACTGGACACACATACAGTG-3’ (forward)  5’-CTGAGGATCTCTGGTTGTGGT-3’ (reverse) |
| ZEB1: | 5’-TTACACCTTTGCATACAGAACCC-3’ (forward)  5’-TTTACGATTACACCCAGACTGC-3’ (reverse) |
| ZEB2: | 5’-GCGATGGTCATGCAGTCAG-3’ (forward)  5’-CAGGTGGCAGGTCATTTTCTT-3’ (reverse) |
| CyclinA2: | 5’-CGCTGGCGGTACTGAAGTC-3’ (forward)  5’-GAGGAACGGTGACATGCTCAT-3’ (reverse) |
| CyclinB1: | 5’-TTGGGGACATTGGTAACAAAGTC-3’ (forward)  5’-ATAGGCTCAGGCGAAAGTTTTT-3’ (reverse) |
| CyclinD1: | 5’-CAATGACCCCGCACGATTTC-3’ (forward)  5’-CATGGAGGGCGGATTGGAA-3’ (reverse) |
| CyclinE1: | 5’-AAGGAGCGGGACACCATGA-3’ (forward)  5’-ACGGTCACGTTTGCCTTCC-3’ (reverse) |
| CDK2: | 5’-AGCCAGAAACAAGTTGACGGGAGA-3’ (forward)  5’-AAGAGGAATGCCAGTGAGAGCAGA-3’ (reverse) |
| CDK4: | 5’-TCAGCACAGTTCGTGAGGTG-3’ (forward)  5’-GTCCATCAGCCGGACAACAT-3’ (reverse) |
| CDK6: | 5’-CCAGATGGCTCTAACCTCAGT-3’ (forward)  5’-AACTTCCACGAAAAAGAGGCTT-3’ (reverse) |
| p27: | 5’-AGTCCATTTGATCAGCGGAGACTCG-3’ (forward)  5’-TCGCACGTTTGACATCTTTCTCCC-3’ (reverse) |
| GAPDH: | 5’-AAGGTCGGAGTCAACGGATTTGGT-3’ (forward)  5’-AGTGATGGCATGGACTGTGGTCAT-3’ (reverse) |
| RBM8A: | 5’-CCACAACGCTCTGTTGAAGG-3’ (forward)  5’-TCTGCGAATTTGTCGTGTATGTC-3’ (reverse) |
| ANG: | 5’-CAAGGCCATCTGTGAAAACAAG-3’ (forward)  5’-CAGGGGGAACCTCCATGTAG-3’ (reverse) |
| TYRO3: | 5’-CAGCCGGTGAAGCTCAACT-3’ (forward)  5’-TGGCACACCTTCTACCGTGA-3’ (reverse) |
| BANP: | 5’-TCGGCAGAACACCATTGTGG-3’ (forward)  5’-GTTCAGGGTGATGAGCGTGAC-3’ (reverse) |
| MUC4: | 5’-GGAGAGGTATCGCCCTGATAG-3’ (forward)  5’-CCGGTGTAGCCTGTAGAACTG-3’ (reverse) |
| SPAG9: | 5’-CAAGCACTCCCACCAAAGG-3’ (forward)  5’-CCCGACCCATTCCTAGTAAATCT-3’ (reverse) |
| ZFP36: | 5’-GACTGAGCTATGTCGGACCTT-3’ (forward)  5’-GAGTTCCGTCTTGTATTTGGGG-3’ (reverse) |
| KCNN4: | 5’-AAGCTCCGGGAACAAGTGAAC-3’ (forward)  5’-CGCCAGCGTGTCAATCTGT-3’ (reverse) |
| PKIB: | 5’-GAGTCTGGGGTCGCCAATTTT-3’ (forward)  5’-TGAACTCTGGATGTCTGGTAAGG-3’ (reverse) |
| SNX16: | 5’-TTCCAGGTTTTCGACTAGCAC-3’ (forward)  5’-AGGCAGTTAGCAATGTCCTTG-3’ (reverse) |

**ChIP**

**For ChIP-quantitative PCR (ChIP-qPCR), primers used are shown as following:**

| Region1: | 5’-CCCTCCAGGGGATGATGCCCGGA-3’ (forward)  5’-GGAGTCTCGGCCGTCACAGCAG-3’ (reverse) |
| --- | --- |
| Region2: | 5’-GGAGTCTCGGCCGTCACAGCAG-3’ (forward)  5’-CACGAGCAAGAGCTGACAGA-3’ (reverse) |
| Region3: | 5’-CACGAGCAAGAGCTGACAGA-3’ (forward)  5’-CAGTGAGCTGAGATGGTGCCACT-3’ (reverse) |
| Region4: | 5’-CAGTGAGCTGAGATGGTGCCACT-3’ (forward)  5’-TATCCGCATTATTATGGCGT-3’ (reverse) |
| Region5: | 5’-TATCCGCATTATTATGGCGT-3’ (forward)  5’-GTCTTGAGGAATCAGAAGCAGA-3’ (reverse) |
| Region6: | 5’-GTCTTGAGGAATCAGAAGCAGA-3’ (forward)  5’-GTCGCTTCCTCCTACCGAGGTTCTG-3’ (reverse) |
| Region7: | 5’-GTCGCTTCCTCCTACCGAGGTTCTG-3’ (forward)  5’-GCCACGCGTCTGGAGTCTGCC-3’ (reverse) |
| Region8: | 5’-GCCACGCGTCTGGAGTCTGCC-3’ (forward)  5’-AGTGCATTTCTGTTCCTGCCCCTC-3’ (reverse) |
| Region9: | 5’-AGTGCATTTCTGTTCCTGCCCCTC-3’ (forward)  5’-CTCAGCTCAGGCTGATGAGAA-3’ (reverse) |
| Region10: | 5’-CTCAGCTCAGGCTGATGAGAA-3’ (forward)  5’-ACACTGCAGCTGCTGGGCCGT-3’ (reverse) |
