# Supplementary figures and images for: PRDM16 functions as a suppressor of lung adenocarcinoma metastasis
Source: J Exp Clin Cancer Res. 2019 Jan 25;38:35. doi: 10.1186/s13046-019-1042-1 (PMC6347838; doi:10.1186/s13046-019-1042-1)

**a**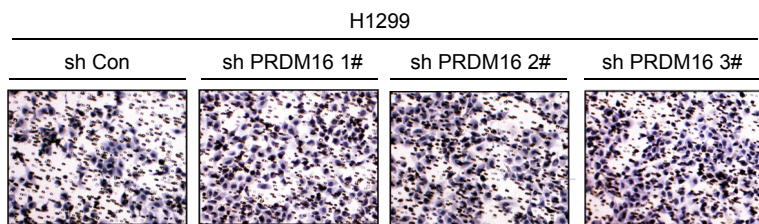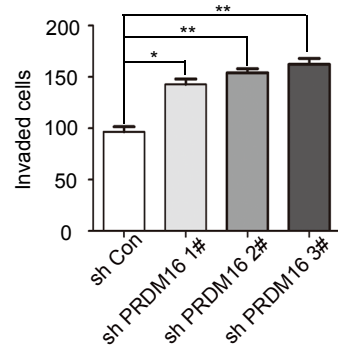**b**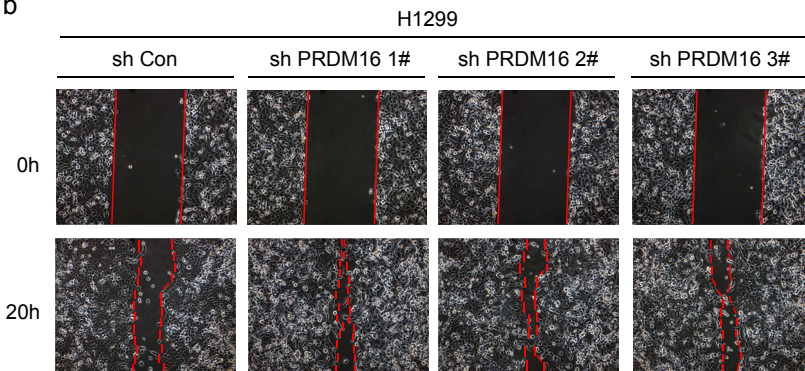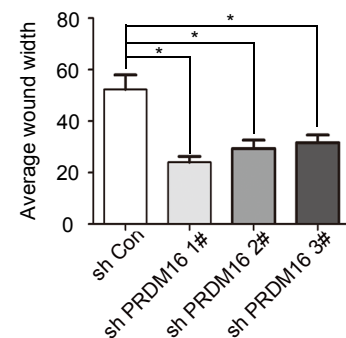**c**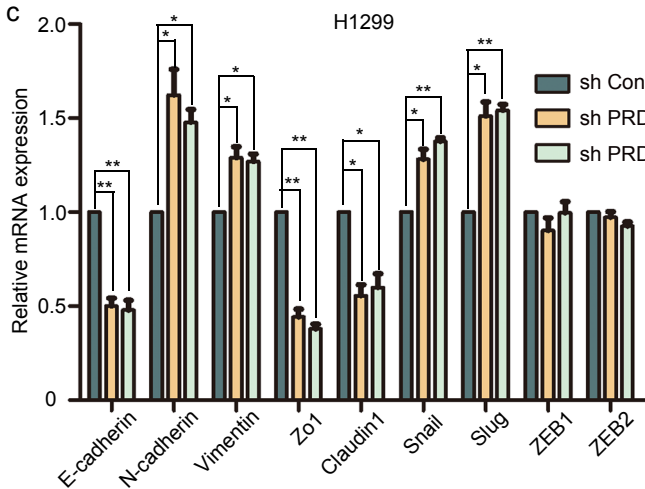**d**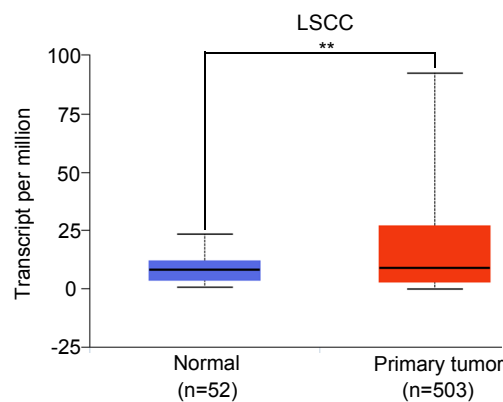**e**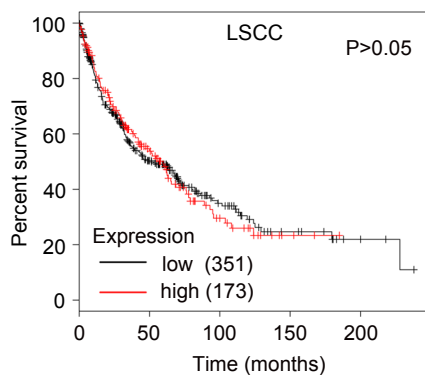**f**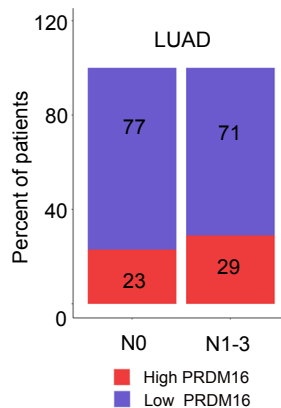**g**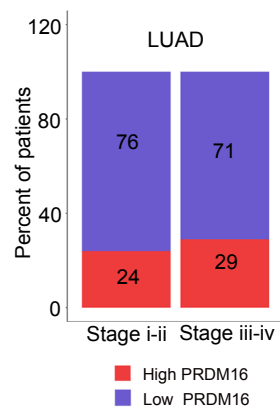

Supplement: Supplementary file 2 — Figure S1. (ZIP 4948 kb) [file 13046_2019_1042_MOESM2_ESM.zip › Additional file 1.pdf]
