# Supplementary material for: PRDM16 functions as a suppressor of lung adenocarcinoma metastasis
Source: J Exp Clin Cancer Res. 2019 Jan 25;38:35. doi: 10.1186/s13046-019-1042-1 (PMC6347838; doi:10.1186/s13046-019-1042-1)
Supplement: Supplementary file 2 — Figure S1. (ZIP 4948 kb) [file 13046_2019_1042_MOESM2_ESM.zip › Additional file 2.doc]

**Additional file 2: Figure S1.** **a** and **b**, Images and quantitation of matrigel invasion assays or wound healing assays of H1299 treated with indicated plasmids. **c**, RT-qPCR analysis of expression of key EMT-related markers in H1299 cells treated with indicated plasmids. **d**, Transcriptional levels of MUC4 in lung squamous cell carcinomas compared with normal lung tissues. Data were obtained from the UALCAN database [25]. **e**, Kaplan-Meier plots of overall survival of patients with lung squamous cell carcinoma stratified by MUC4 expression. Data were obtained from the Kaplan-Meier plotter database [28]. **f** and **g**, Percentage of lung adenocarcinoma patients with high expression and low expression of MUC4 stratified according to lymph node status or TNM stage (n=497).
